# Supplementary material for: Exploring red cell distribution width as a potential risk factor in emergency bowel surgery—A retrospective cohort study
Source: PLoS One. 2022 May 5;17(5):e0266041. doi: 10.1371/journal.pone.0266041 (PMC9071152; doi:10.1371/journal.pone.0266041)
Supplement: S1 Annex — (DOCX) [file pone.0266041.s002.docx]

**Annex – statistical discussion**

**Background:**

The primary aim of our analysis was to investigate the association between RDW and mortality in emergency laparotomy patients at St Mary’s Imperial College Healthcare NHS Trust. We also explored the added prognostic value of RDW for 30-day and overall mortality. To achieve this, we first compared the average RDW values between survivors and non-survivors. Using a Cox proportional hazards design, we then examined the predictive value of RDW by developing two separate nested models for both mortality outcomes. Our secondary objective was to establish whether RDW is associated with frailty in NELA patients. To obtain an initial estimate of frailty in our dataset we carried out a descriptive analysis. We then went on to design a binary logistic regression model to describe patterns of frailty based on RDW.

All data analysis was performed in R (version 3.5.2) using the following packages: *car, DescTools,* *dplyr, ggplot2, glmpath, gridExtra, Hmisc, rms, survival,* and *survminer* [1-11].

**Study population:**

The inclusion criteria for our study matched those published by the National Emergency Laparotomy Audit (NELA) [12].

**Data collection:**

Data were pseudo anonymised using the unique NELA identifier. Handling of online NELA data entered by individual NHS trusts adheres to strict information governance standards, which are laid out on the NELA website [13]. All additional administrative or clinical data required were collected and analysed in accordance with the General Data Protection Regulation.

We cross-referenced NELA mortality data with our institution’s clinical information system (Cerner Millennium®, Cerner Corporation, MI, USA), which interlinks with The Spine (NHS Digital, NHS England) integrating summary care records and patient demographic data, thereby maximising follow-up data capture.

Only the index surgery was taken into account for this study. Subsequent laparotomies on the same patient as part of their inpatient stay were not analysed. No patient was discharged following emergency bowel surgery and required a second emergency laparotomy at a later date.

The NELA risk adjustment model captures a series of routinely collected risk factors. These can be summarised as demographic (sex, age), preoperative (ASA score, surgical urgency, admission heart rhythm, number of operative interventions, pre-existing cardiac or respiratory disease), physiological (blood pressure, heart rate, Glasgow coma score, creatinine, sodium, potassium, urea, white blood cells), and operative findings [14]. Thus, the NELA risk score requires clinicians to estimate peritoneal soiling, operative severity, blood loss and the extent of suspected malignancy preoperatively [14]. While these measures are not preoperative in the strictest sense, we used the values estimated by clinicians. Overall missing data for these values was small and intraoperative findings were used. The proportion of missing data was 1.11% (n=4) for peritoneal soiling, 0.55% (n=2) for operative severity, 0.55% (n=2) for blood loss and 0.27% (n=1) for severity of malignancy. We thought this to be the most acceptable compromise without resorting to multiple imputations in a small dataset, while simultaneously avoiding the loss of information seen with complete case analysis [15].

In our study, we understood preoperative to mean the first set of laboratory values on admission. For existing non-general surgery inpatients requiring emergency laparotomy, we used blood test results twenty-four hours before surgery. Although an arbitrary cut-off we aimed to mirror the perioperative approach (reducing pre-surgery resuscitative confounders) taken for acute surgical admissions.

Finally, the concept of frailty in emergency general surgery is well established [16]. A variety of tools are used to measure frailty and consequently any validated instrument was accepted for the purpose of this study. Failure to document the presence or absence of frailty resulted in a review of the medical notes. Evaluating the admission clerking for clinical descriptors used by the Clinical Frailty Scale (CFS) was thought to be a satisfactory alternative and has been used previously [16].

**Survival analysis – Cox proportional hazards regression modelling**

**Outcomes:**

Outcomes analysed were 30-day and overall mortality rates following laparotomy. Baseline event rate graphs are shown in Figure 1. To compare the event rate curves across RDW quartiles, we used the log-rank test (Figure 2 main article). A fundamental assumption of the log-rank test is that the risk of an event in one group relative to the other does not change with time. The proportional hazards assumption is violated when mortality rate curves cross, and the log-rank test should not be used [17]. Hence, we did not use it to analyse 30-day mortality rates across RDW strata. Advanced statistical techniques exist to compare survival curves with time-dependent covariates (e.g., the Combined test) but are beyond the scope of this text [18]. Furthermore, it seems clinically unlikely that the exposure effect for a single RDW quartile varies with time. A more plausible explanation is that the multiple overlaps seen in RDW4 arise because the surviving cohort size is small enough for random crossings to occur.


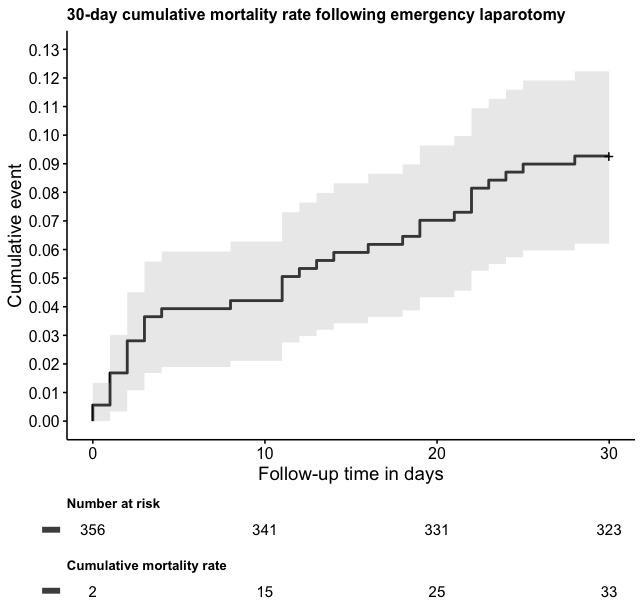

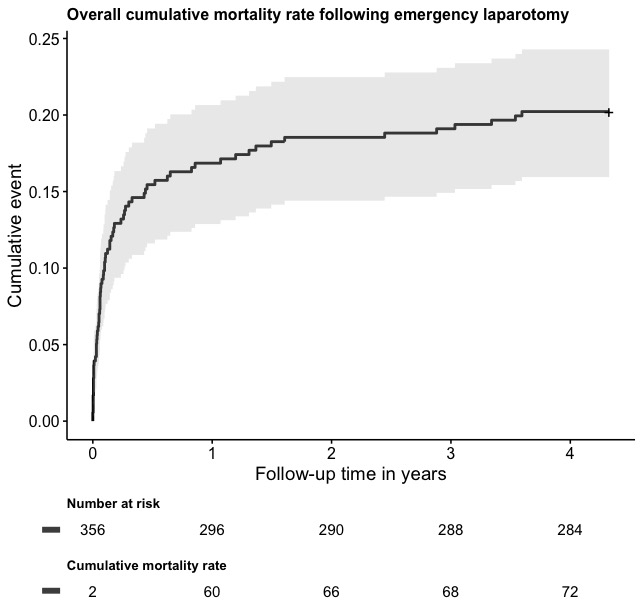


**Figure 1** Cumulative mortality rate plots for 30-day and total follow-up mortality. At 30-days post-emergency laparotomy, cumulative mortality is 9.3% compared to 20.2% overall. The shaded areas represent 95% confidence intervals.

**Outliers:**

Outliers can significantly affect regression modelling. Continuous data were visualised using boxplots (Fig 2). No outliers were thought to be due to data handling errors, and all were clinically plausible, thus winsorisation at the 5^th^ or 95^th^ centile was carried out. Table 1 summarises the winsorised ranges.

**
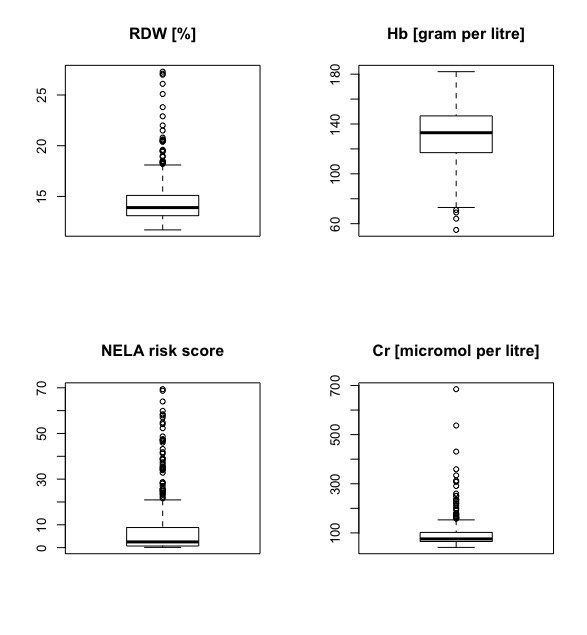
**

**Figure 2** Boxplots of continuous predictor variables. RDW: red cell distribution width,

Hb: haemoglobin, NELA: National Emergency Laparotomy Audit, Cr: creatinine

| **Table 1** Winsorised ranges of continuous predictors | | | | |
| --- | --- | --- | --- | --- |
|  |  | |  |  |
| **Physiological risk factor** | | **Median** | **Interquartile range** | **Winsorised range** |
| Red cell distribution width (RDW %) | | 13.9 | 13.1-15.1 | 11.7-19.5 |
| Haemoglobin (Hb gl^-1^) | | 133 | 117-146.2 | 86-182 |
| NELA risk score | | 2.5 | 0.7-8.8 | 0.1-41.4 |
| Creatinine (Cr μmoll^-1^) | | 75.5 | 65-102 | 41-202 |
| NELA: National Emergency Laparotomy Audit | | | | |

**Model predictors**

We did not assume linearity of effect for any of the continuous predictors. Variables were expanded by fitting three-knot restricted cubic spline functions followed by ANOVA tests to assess for significant non-linearity. This was done for the 30-day and overall mortality models.

We used restricted cubic splines because they allow transformation of a continuous predictor, and provide a simple way to create, test and fit non-linear relationships in regression models. Moreover, it avoids categorising continuous variables, which significantly reduces their predictive power [19].

Spline functions are piecewise polynomials used in curve fitting. This approach divides the range of values of a predictor utilising a set of knots. Polynomials are then fitted between the knots. Pragmatically, cubic splines are used as they are the smallest degree polynomial that provides sufficient flexibility fitting the data, while not requiring too many degrees of freedom [19, 20]. However, cubic splines tend to fit the data poorly before the first and after the last knot, the so-called tails. Therefore, constraining the function to be linear in the tails tends to provide a better fit for the data [19]. This restricted cubic spline function has the additional benefit of reducing the degrees of freedom needed, which is particularly relevant in small studies like ours [19, 20].

Occasionally, the number and position of knots may be suggested by theory. However, in most situations, there is no way to pre-specify knots. Crucially, it has been shown that the location of knots in a restricted cubic spline model is far less critical than their number [19, 20]. Placing knots at fixed quantiles of a continuous predictor is thought to be a reasonable approach for most datasets [20]. Selecting the number of knots is dictated by the sample size available and the desired flexibility of predictor fit. In smaller sample sizes, three knots should be used to have enough points in each interval to estimate the cubic polynomial [19]. Thus, in our study, we used three-knot restricted cubic splines with default quantiles at 0.1, 0.5 and 0.9 (default values used in rms package).

Haemoglobin and RDW demonstrated significant non-linearity on testing. For haemoglobin, we used a three-knot restricted cubic spline. RDW closely resembled a log fit, and we chose a logarithmic transformation saving degrees of freedom in the process (Figure 3). No other predictors were significantly non-linear. Categorical predictors were expanded using dummy variables. All transformations are summarised in Table 2.


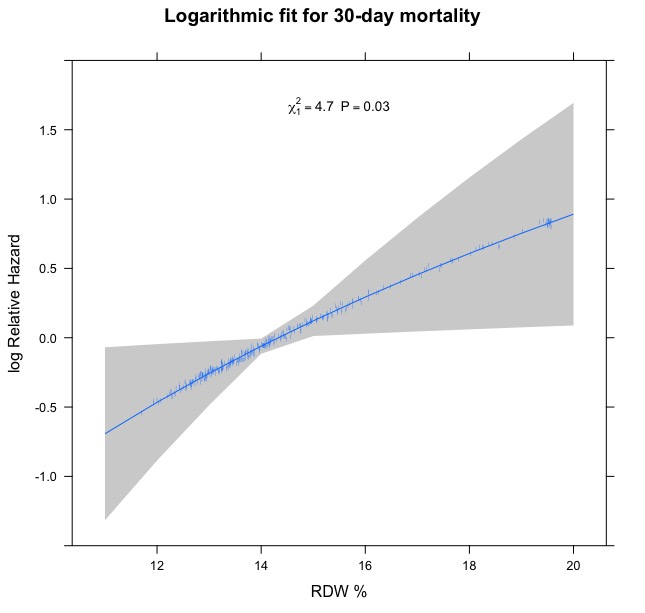

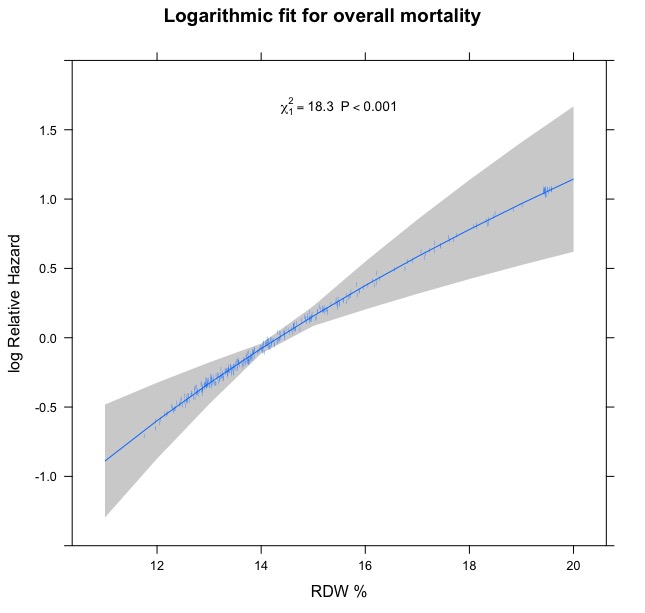


**Figure 3** Logarithmic transformation for red cell distribution width (RDW) for 30-day and overall mortality

| **Table 2**  Description of continuous predictor variables for the Cox regression models | | | |
| --- | --- | --- | --- |
| Variable | Type | Transformations for 30-day mortality model | Transformations for overall mortality model |
|  |  |  |  |
| **RDW** | Continuous | Logarithmic | Logarithmic |
| **NELA risk score** | Continuous | Linear | Linear |
| **Haemoglobin** | Continuous | Restricted cubic spline with 3-knots | Restricted cubic spline with 3-knots |
| **Creatinine** | Continuous | Linear | Linear |
| RDW: Red cell distribution width, NELA: National Emergency Laparotomy Audit | | | |

**Multicollinearity**

Multicollinearity occurs when two or more exposure variables are highly correlated. The presence of multicollinearity can adversely affect the results in a regression model. The standard errors of the regression coefficient estimates become inflated, and corresponding tests have reduced power [19]. The variance inflation factor (VIF) is often used to detect multicollinearity in regression modelling. A value of one means that the predictor is not correlated with other variables. There remains some debate around what constitutes a large VIF. As a rule of thumb, a VIF above ten is thought to indicate high correlation and hence to be problematic [21]. Occasionally, a high VIF is of little concern. This is particularly relevant when fitting cubic splines where the component variables can be very collinear. However, this is unlikely to cause problems as the component variables are connected algebraically [19]. Equally, high VIFs for dummy variables representing categorical predictors are not usually an issue [19]. We tested all predictors across both of our models for multicollinearity. Variance inflation factors ranged from 1.23-6.37. As expected, higher VIFs were found for haemoglobin, which was modelled using a restricted cubic spline (range 5.31-6.37) and the categorical variable laparotomy indication (range 2.27-5.41). The continuous variables RDW, creatinine and NELA risk score had low VIF values (range 1.23-1.34). Consequently, it was felt that no significant multicollinearity was present.

**Interactions**

An exhaustive search for interactions with all possible variables is rarely useful and is often limited by the sample size [22]. Instead, we calculated overall pooled estimates for each individual exposure. Statistically significant and clinically relevant interactions were then pursued. Consequently, providing a simplified but practical picture of reality, while avoiding a model with too many additional parameters and thus loss of power.

In both nested models, the pooled test was significant for haemoglobin and creatinine. Hence, we included this interaction term for both models. A possible interpretation could be that a high creatinine in the context of anaemia is likely to be chronic. Therefore, conferring conceivably less risk than an acute rise on the background of a normal haemoglobin.

Creatinine, as a surrogate marker for renal function, interacts with the NELA risk score in the overall mortality model. Although creatinine is part of the composite NELA score, individually, it influences risk differently. For example, high levels of creatinine (e.g., 200 μmoll^-1^) confer an almost identical risk irrespective of the patient’s NELA score in our model (Fig. 3 main article). Clinically suggesting that renal failure is always a poor prognostic sign. Conversely, normal renal function (e.g.,75 μmoll^-1^) needs to be interpreted in the context of the NELA risk score.

**Proportional hazards assumption**

In Cox regression analysis, the proportional hazards assumption is a central tenet. We examined both our nested models globally and plotted Schoenfeld residuals for all variables individually. Global p values for the 30-day mortality model were 0.662 without RDW and 0.192 with RDW. For the overall mortality model, p values were 0.561 and 0.146, respectively. Also, none of the individual variables in any of the models was significant, confirming the proportional hazards assumption.

**Influential observations**

Ideally, every observation should influence the fit of a regression model. However, with a small sample size, a significant treatment effect or the shape of a regression effect may rest only on a few observations. These overly influential observations also lead to increased variance of the predicted values [19]. An observation may be regarded as influential if it exerts more than its fair share in determining the values of the regression coefficients. Harrell’s rms package can screen regression models for influential observations using DFBETAS [9]. Here, the standardised change in regression coefficients upon leaving out each observation in turn, determines which observations are influential. The default cut-off is 0.2, indicating a change in a regression coefficient of 0.2 standard errors [9,19].

In the 30-day mortality model without RDW, a total of 35 (9.8%) observations had a DFBETA ≥$\pm$0.2. Whereas in the model with RDW 36 (10.1%) points were influential. Similarly, for the overall model, 39 (11%) and 37 (10.4%) observations respectively, were significant. For the variable RDW, five cases (1.4%) had a DFBETA ≥0.2 in the 30-day mortality regression model, and four (1.1%) were influential in the overall model. A sensitivity analysis excluding these cases demonstrated minimal effect. For the 30-day mortality model, global χ^2^ decreased from 64.21 to 60. For the overall model, global χ^2^ increased from 78.05 to 80.79.

**Describing predictor effects**

Unlike linear functions describing predictor effects, including non-linear terms and interactions, is not straightforward. Here we plot the effects for all predictors in the nested 30-day and overall mortality models (Figure 4). In these plots, the interaction terms have been incorporated into the variables’ relative importance. Note that both models have insignificant predictors. These are not deleted, as that would not improve predictive accuracy, and it would make accurate confidence intervals hard to obtain [19].


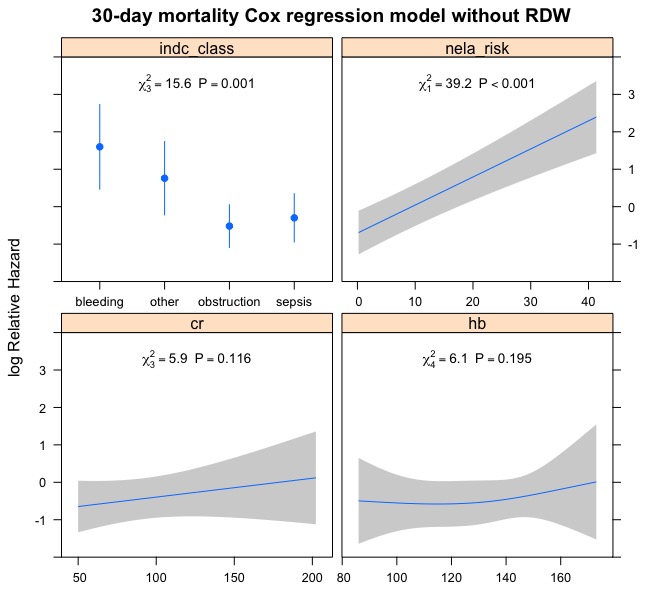

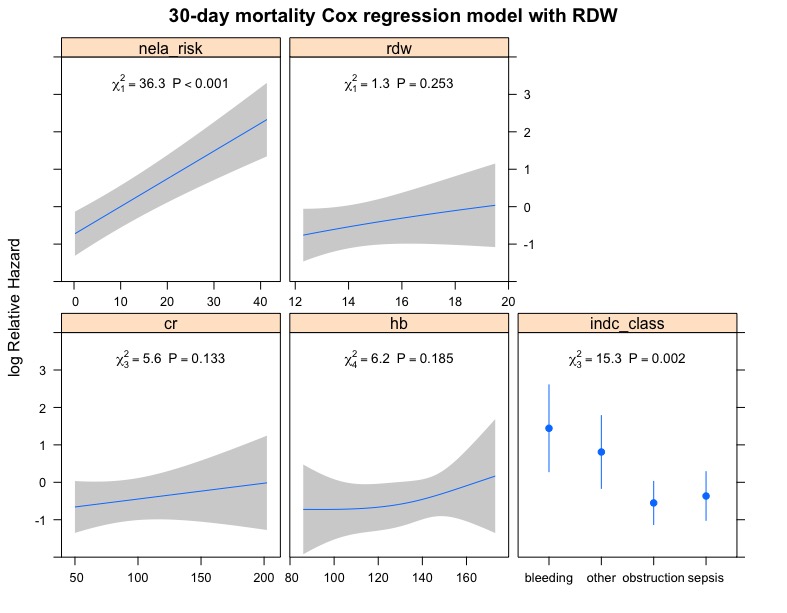


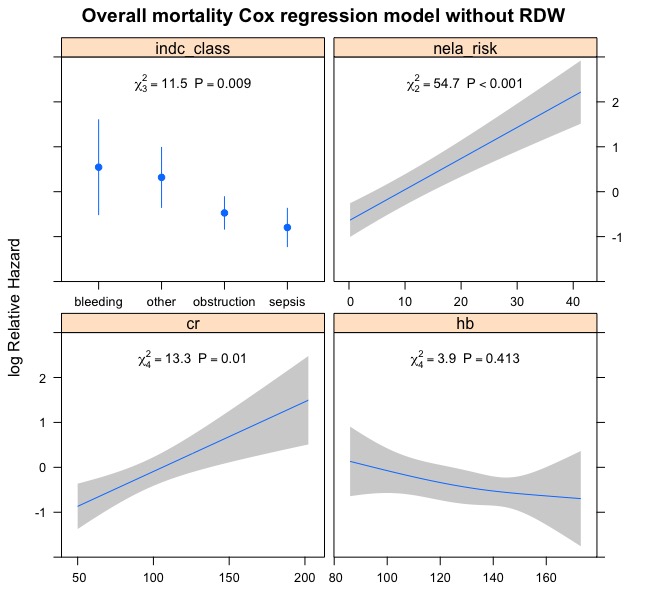

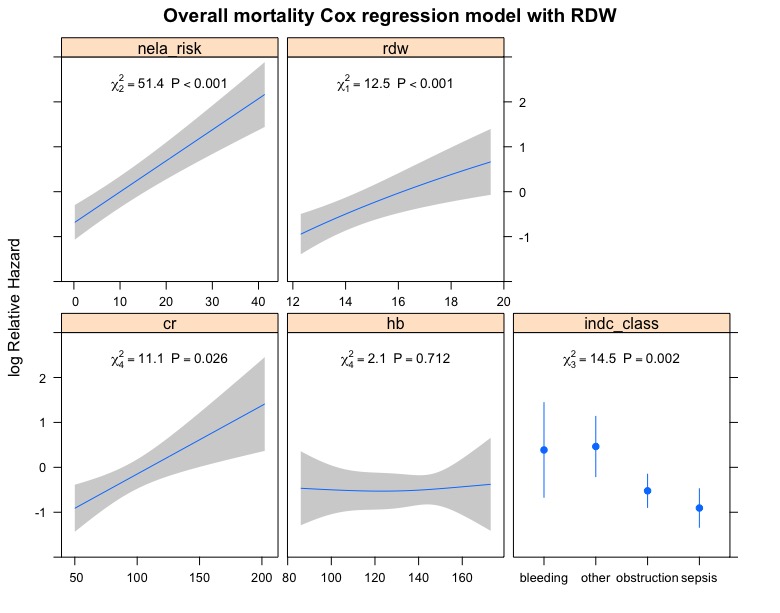


**Figure 4** The shape of each predictor on the log hazard of death for both nested 30-day and overall mortality models is displayed. Pointwise 0.95 confidence bands are also shown. All Y-axes have the same scale, making it easy to see which predictors are strongest. cr: creatinine (μmoll^-1^), hb: haemoglobin (gl^-1^), ind_class: indication for laparotomy, nela_risk: NELA risk score, rdw: red cell distribution width (%).

**Added value of RDW**

We set out to assess the amount of new information provided by RDW. Here the notion of added value is in the predictive sense. For both our nested models, we performed a Likelihood ratio (LR) χ^2^ test. The added value of RDW is obtained by comparing log-likelihoods. We did this for both 30-day and overall mortality. The LR test resulted in Δχ^2^ (1, N=356) = 1.27, p=0.260 for the 30-day mortality model. The test statistics for the overall model were as follows: Δχ^2^ (1, N=356) = 11.53, p<0.001. From the last test, there is evidence that RDW adds some prognostic value. RDW adds no new information for 30-day mortality. This is mirrored by its minimal relative contribution, as demonstrated in Figure 5.

To assess how much new predictive information RDW adds, various indices can be used. Unlike LR χ^2^, these indices are sample size-independent and do not require categorisation of data. We used the fraction of new information, which is the proportion of total predictive information that was added by RDW (1- [LR χ^2^ base model/ LR χ^2^ full model]). Thus, the fraction of total prognostic information that was due to RDW is estimated to be 0.14 (Table 3) [23].

**
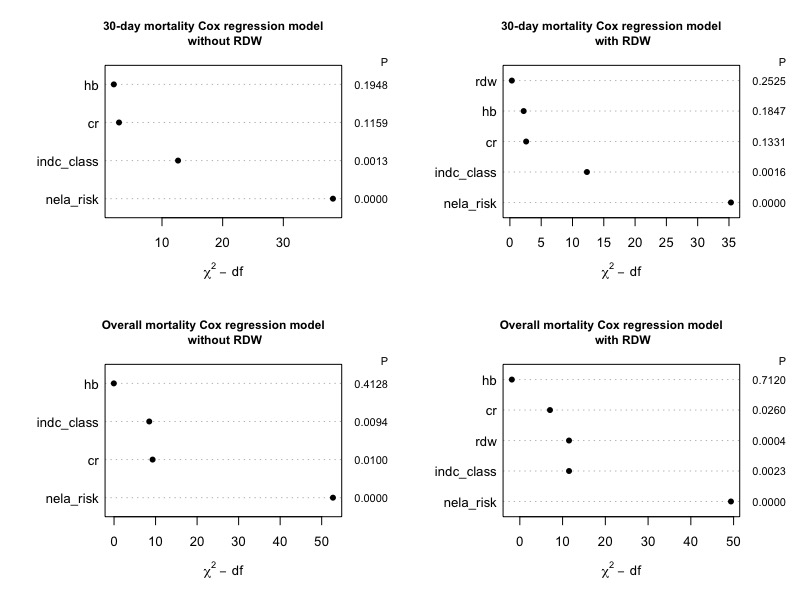
**

**Figure 5** Relative importance of predictors for both nested models are plotted as Wald χ^2^ statistics, penalised for degrees of freedom (df), in descending order. Interaction terms and non-linear effects are included in the variables. Higher χ^2^ – df values indicate stronger association. Hb: haemoglobin, cr: creatinine, indc_class: indication for laparotomy, nela_risk: NELA risk score

| **Table 3** Added valued of RDW | | | | |
| --- | --- | --- | --- | --- |
|  | | | | |
|  | | Model without RDW | Model with RDW | P value |
|  | | | | |
| **30-day mortality**  **Cox regression model** | | | | |
| LR χ^2^ | 58.67 (9 df) | | 59.94 (10 df) | 0.260 |
| Nagelkerke R^2^ | 0.230 | | 0.235 |  |
|  | | | | |
| **Overall mortality**  **Cox regression model** | | | | |
| LR χ^2^ | 68.36 (10 df) | | 79.89 (11 df) | <0.001 |
| Nagelkerke R^2^ | 0.193 | | 0.223 |  |
| Fraction of new information |  | | 0.144 |  |
| The fraction of new information is the proportion of total predictive information that was added by RDW. It can be calculated by LR χ^2^ (base model)/LR χ^2^ (full model) and subtracting the result from 1. df: degrees freedom, LR χ^2^: likelihood ratio test statistic (distributed χ^2^). | | | | |

**Validating the model**

In order to ascertain the predictive ability of our full overall mortality model (with RDW), we assessed both discrimination (how well the model picks out low and high-risk patients) and calibration (the agreement between the observed and predicted outcomes). This process is known as model validation. Ideally, the model should be validated externally, using patient data not used for model development. However, in practice, most models are validated internally using for example bootstrapping.

Bootstrapping allows a new dataset of the same size to be generated from the original study cohort by resampling with replacement. In turn, this new dataset is used to derive a model, which is then applied without change to the original sample. The difference in accuracy between the bootstrap sample and the original sample is called optimism [19]. Subsequently, this process is repeated many times over in order to obtain an average optimism. This mean optimism is subtracted from the apparent accuracy of the original model, thereby generating optimism-corrected (also called overfitting-corrected) estimates [19]. Therefore, bootstrapping is one way to provide a reasonable assessment of future model performance.

Consequently, we validated our overall mortality Cox regression model for Somers’ D_xy_ rank correlation between predicted log hazard and observed survival time, and for slope shrinkage. The bootstrap is used (with 1000 resamples) to penalise for possible overfitting, as discussed above. The results are shown in Table 4.

| **Table 4**  Bootstrap (1000 resamples) validation of the full model for overall mortality | | | | | | |
| --- | --- | --- | --- | --- | --- | --- |
| Index | Original sample | Training sample | Test sample | Optimism | Corrected index | n |
| D_xy_ | 0.5607 | 0.5848 | 0.5253 | 0.0595 | 0.5013 | 1000 |
| C-index | 0.7804 | 0.7924 | 0.7627 | 0.0297 | 0.7507 | 1000 |
| R^2^ | 0.2226 | 0.2512 | 0.1924 | 0.0587 | 0.1639 | 1000 |
| Slope | 1.0000 | 1.0000 | 0.8336 | 0.1664 | 0.8336 | 1000 |
| D_xy_: Somers’ D_xy_ rank correlation, C-index: concordance probability (C=[D_xy_+1]/2), R^2^: Nagelkerke R^2^, slope:  refers to the calibration slope of the model – the discrimination in the validation set is the same when the slope =1 (if the slope is <1 discrimination is worse) | | | | | | |

In Table 4 *training* refers to the accuracy when the model is derived from the bootstrap sample, and *test* relates to the accuracy when this is applied without modification to the original sample. The apparent D_xy_ is 0.56 (C-index = 0.78) but a better estimate of how well the model will discriminate in the future is D_xy_ = 0.50 (C-index = 0.75). The corrected slope is 0.83 and can be thought of as a shrinkage factor that takes overfitting into account [24]. Expressed differently, approximately 0.17 of the model fitting will be noise, especially in regard to calibration accuracy. Accordingly, bootstrap calibration curves demonstrate some overfitting (Figure 6).

**
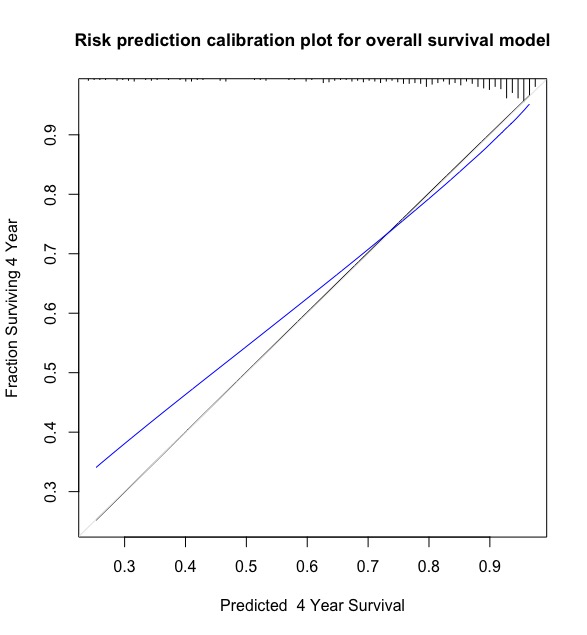
**

**Figure 6** Bootstrap estimate of calibration accuracy for the full overall mortality model. The black line corresponds to the apparent predictive accuracy of the overall survival model and overlies the grey ideal line. The blue curve corresponds to bootstrap-corrected estimates, demonstrating some overfitting. The absolute error is particularly appreciable when 4-year survival is predicted to be low. A predicted 4-year survival of about 15% translates to an observed survival of approximately 35%.

**Sample size, overfitting and limits on the number of predictors**

Our main aim was not to develop a new and clinically valid prediction model but to generally explore the added prognostic value of RDW in emergency laparotomy patients. Thus, how well future observed values agree with predicted ones, was of lesser importance. Nonetheless, we felt that the amount of overfitting warranted some adjustment.

Typically, in overfitted models, low predictions will be too low and high predictions too high (Figure 6). One of the reasons why risk prediction tools perform less well in future populations is because the fitted model captures not only signal but also the random variation (noise) in the development dataset [19].

This is particularly true when the ratio of the number of events (here deaths) to the number of parameters is small [24]. The events per variable (EPV) ratio thought to avoid overfitting is ten or more and is often used to determine sample size [24]. Our full overall mortality model has an EPV of 72/11=6.55, well below the minimum of ten. Note that the number of candidate variables should include all variables screened for association with the response, including non-linear terms and interactions. Hence, to improve the future predictive performance of our model, we used both data reduction and shrinkage [19].

Informal data reduction was carried out for all models prior to their development. The base models are simplified and contain only the powerful NELA risk score, haemoglobin, creatinine and the indication for laparotomy. Including the NELA score incorporates many of the risk factors that are routinely collected in clinical practice and validated in a large population-based data set, therefore saving many degrees of freedom. Table 5 summarises the preliminary data reduction strategy, with a total saving of five degrees of freedom. Including the interactions for the full overall mortality model brings the degrees of freedom back to eleven.

| **Table 5**  Initial and final allocation of degrees of freedom (d.f.) | | | | | |
| --- | --- | --- | --- | --- | --- |
| **Predictor** | **d.f.** | **Original levels** | **Reductions** | **d.f. saved** | |
| NELA risk score^†^ | 2 |  | No significant non-linearity | | 1 |
| Haemoglobin | 2 |  | Use three knots | |  |
| Creatinine | 2 |  | No significant non-linearity | | 1 |
| Indication for laparotomy | 5 | Obstruction, sepsis, bleeding, ischaemia, colitis & other | Ischaemia & colitis combined  into other | | 2 |
| RDW | 2 |  | Logarithmic fit | | 1 |
| ^†^ Including every variable of the NELA risk model would hugely inflate the d.f. of the models; hence the composite risk score was chosen. Continuous predictors are expanded by fitting three-knot restricted cubic spline functions, which contain one non-linear term and have a total of 2 d.f. ANOVA tests were used to determine if there was a significant non-linear component. NELA: National emergency laparotomy audit, RDW: Red cell distribution width | | | | | |

Another way to minimise the problem of overfitting is to use statistical estimation methods that shrink the regression coefficients towards zero. Shrinkage reduces the range of predicted risks and brings them closer to the average risk, thereby resulting in more accurate predictions when the model is applied to future populations [19,24]. We chose the least absolute shrinkage and selection operator (LASSO) to penalise our model for overfitting.

LASSO uses the sum of absolute values of regression coefficients for penalisation of model complexity. It shrinks each coefficient toward zero by a constant factor, truncating at zero. LASSO regression was preferred as it performs well in situations where some of the predictors have large coefficients (e.g., RDW 3.7187) and others have small coefficients (e.g., NELA risk score 0.1053) [25]. In contrast to Ridge regression, which will perform better when the outcome is a function of many predictors, all with coefficients of roughly equal size [25]. In our full overall mortality model, none of the coefficients was shrunk to zero, and the mean shrinkage was 1.02.

**Frailty analysis – Binary logistic regression modelling**

Baseline descriptive statistics for the frailty cohort are presented in Table 6. For 216 (60.7%) patients, an assessment of frailty was either not documented or not possible from reviewing the admission notes. Failure to appropriately account for this large amount of missing data in later analyses can lead to bias. Accordingly, to draw any valid inferences in the presence of missing data, it is essential to understand the patterns of missing values. Figure 7 demonstrates which variables tended to be missing, as well as highlighting the proportion of missing values for each predictor.

| **Table 6.** Patient characteristics of patients undergoing emergency laparotomy unadjusted frailty rates. | | | |
| --- | --- | --- | --- |
| *n*=140 | Frail | Not Frail | *P* value |
|  | *n*=24 (17.1%) | *n*=116 (82.9%) |  |
| **Demographic** |  |  |  |
| Median age in years | 75 (59-81) | 56 (44-75) | 0.01 |
| Female sex (%) | 13 (54.2) | 61 (52.6) | 1 |
| **Preoperative** |  |  |  |
| Median NELA predicted 30-day mortality (%) | 12.4 (5.5-34.2) | 2.3 (0.6-6.7) | <0.001 |
| ASA ≥3 (%) | 22 (91.7) | 50 (43.1) | <0.001 |
| Urgency of surgery |  |  | 0.12 |
| Expedited >18 hours (%) | 8 (33.3) | 18 (15.5) |  |
| Urgent 6-18 hours (%) | 5 (20.8) | 41 (35.3) |  |
| Urgent 2-6 hours (%) | 11 (45.8) | 49 (42.2) |  |
| Immediate < 2 hours (%) | 0 | 8 (6.9) |  |
| ECG |  |  | 0.697 |
| No abnormalities (%) | 23 (95.8) | 101 (87.1) |  |
| AF rate 60-90 min^-1^ (%) | 0 | 3 (2.6) |  |
| AF rate >90 min^-1^ or  any other abnormal  rhythm, ST changes (%) | 1 (4.2) | 12 (10.3) |  |
| Cardiac signs |  |  | 0.171 |
| No failure (%) | 17 (70.8) | 98 (84.4) |  |
| Diuretic, digoxin, anti-  anginal or hypertensive  therapy (%) | 5 (20.8) | 15 (12.9) |  |
| Peripheral oedema,  warfarin therapy (%) | 1 (4.2) | 2 (1.7) |  |
| Raised JVP or CXR signs (%) | 1 (4.2) | 1 (0.9) |  |
| Respiratory history |  |  | 1 |
| No dyspnoea (%) | 20 (83.3) | 93 (80.2) |  |
| Dyspnoea on exertion (%) | 3 (12.5) | 16 (13.8) |  |
| Dyspnoea limiting exertion (%) | 1 (4.2) | 5 (4.3) |  |
| Dyspnoea at rest (%) | 0 | 2 (1.7) |  |
| **Clinical values** |  |  |  |
| RDW (%) | 15 (14.2-17.2) | 13.5 (13.0-14.6) | <0.001 |
| Haemoglobin (gl^-1^) | 120 (108-138) | 136 (122-147) | 0.012 |
| Creatinine (μmoll^-1^) | 102 (72-131) | 73 (64-91) | 0.004 |
| Urea (mmoll^-1^) | 7.1 (4.4-12.8) | 5.6 (3.8-8.9) | 0.269 |
| Sodium (mmoll^-1^) | 137 (135-141) | 138 (135-140) | 0.98 |
| WBC (x10^9^l^-1^) | 9.3 (6.6-12.1) | 10.6 (8-14.2) | 0.147 |
| Systolic blood pressure  (mmHg) | 117 (93-131) | 127 (111-138) | 0.056 |
| Pulse (beats min^-1^) | 92 (82-103) | 87 (73-101) | 0.437 |
| **Perioperative** |  |  |  |
| Operative severity |  |  | 0.337 |
| Major (%) | 14 (58.3) | 81 (69.8) |  |
| Major+ (%) | 10 (41.7) | 35 (30.2) |  |
| Peritoneal soiling |  |  | 0.248 |
| None (%) | 16 (66.7) | 53 (45.7) |  |
| Serous fluid (%) | 4 (16.7) | 22 (19) |  |
| Localised pus (%) | 1 (4.2) | 7 (6) |  |
| Free bowel content, pus, or  blood (%) | 3 (12.5) | 34 (29.3) |  |
| Intraoperative blood loss |  |  | 0.678 |
| <100ml (%) | 9 (37.5) | 51 (44) |  |
| 101-500ml (%) | 13 (54.2) | 59 (50.9) |  |
| 501-999ml (%) | 2 (8.3) | 5 (4.3) |  |
| >1000ml (%) | 0 | 1 (0.9) |  |
| Severity of malignancy |  |  | 0.03 |
| None (%) | 17 (70.8) | 105 (90.5) |  |
| Primary only (%) | 4 (16.7) | 5 (4.3) |  |
| Nodal metastasis (%) | 1 (4.2) | 1 (0.9) |  |
| Distant metastasis (%) | 2 (8.3) | 5 (4.3) |  |
| Observed 30-day mortality (%) | 7 (29.2%) | 10 (8.6%) | 0.01 |
| Observed overall mortality (%) | 11 (45.8%) | 17 (14.7%) | 0.001 |
| For 216 patient there was insufficient information to allow an assessment of frailty. Continuous variables are shown as median and interquartile ranges. Categorical variables are shown as a frequency (%). Non-winsorised values were used to draw up the table. *P* values were calculated using Wilcoxon-Mann-Whitney testing or the χ^2^ with Yates continuity correction/Fisher’s exact test (testing for overall difference) as appropriate. AF: atrial fibrillation, ASA: American Society of Anaesthesiologist physical status classification system, CXR: chest radiograph, ECG: electrocardiogram, JVP: jugular venous pulse, Major+: all colonic resections, gastrectomy, laparostomy, intestinal bypass, reoperations for bleeding/sepsis, Major: all other including stoma formation, small bowel resection, adhesiolysis, repair of perforated/bleeding ulcer, NELA: National Emergency Laparotomy Audit, RDW: red cell distribution width, WBC: white blood cell count | | | |


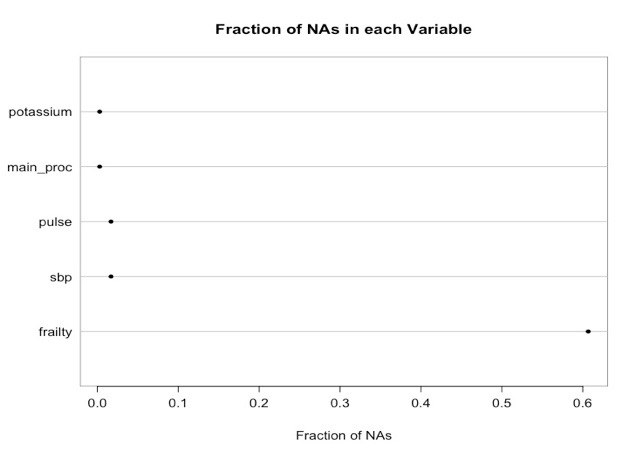

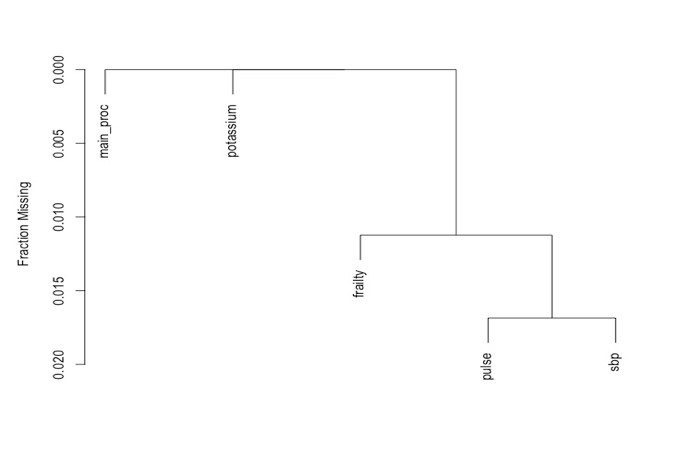


**Figure 7** Patterns of missing data. The left-hand plot shows the fraction of observations missing for each predictor. The right-hand graph illustrates a hierarchical cluster analysis of missingness combinations. The Y-axis is the fraction of observations for which both variables are missing. Main operative intervention (main_proc, 1 missing value), potassium (1 missing value), pulse (6 missing values), systolic blood pressure (sbp, 6 missing values), frailty assessment (frailty, 216 missing values)

Missing NELA database values were cross-referenced with the institution’s clinical information system generating a complete dataset for the exposure variables. In turn, this allowed us to compare baseline characteristics between patients with missing and observed frailty outcomes. Table 7 summarises key features among both groups, demonstrating little difference. Furthermore, there was no significant relationship between death and missing frailty status.

| **Table S7** Comparison between patients with missing and documented frailty status | | | |
| --- | --- | --- | --- |
| N=356 | Missing frailty status | Observed frailty status | p value |
|  | n=216 (60.7%) | n=140 (39.3%) |  |
| **Demographic** |  |  |  |
| Median age in years | 61 (44-72) | 61 (46-78) | 0.2892 |
| Female sex (%) | 110 (50.9) | 74 (52.9) | 0.7455 |
| **Preoperative** |  |  |  |
| Median NELA predicted 30-day mortality (%) | 2.1 (0.8-8) | 3.5 (0.7-11.2) | 0.4917 |
| ASA ≥3 n (%) | 109 (50.5) | 72 (51.4) | 0.9137 |
| Urgency of surgery |  |  | 0.4792 |
| Expedited >18 hours (%) | 40 (18.5) | 26 (18.6) |  |
| Urgent 6-18 hours (%) | 86 (39.8) | 46 (32.9) |  |
| Urgent 2-6 hours (%) | 76 (35.2) | 60 (42.9) |  |
| Immediate < 2 hours (%) | 14 (6.5) | 8 (5.7) |  |
| ECG |  |  | 0.1771 |
| No abnormalities (%) | 193 (89.4) | 124 (88.6) |  |
| AF rate 60-90 min^-1^ (%) | 11 (5.1) | 3 (2.1) |  |
| AF rate >90 min^-1^ or  any other abnormal  rhythm, ST changes (%) | 12 (5.6) | 13 (9.3) |  |
| Cardiac signs |  |  | 0.9699 |
| No failure (%) | 175 (81) | 115 (82.1) |  |
| Diuretic, digoxin, anti-  anginal or hypertensive  therapy (%) | 32 (14.8) | 20 (14.3) |  |
| Peripheral oedema,  warfarin therapy (%) | 4 (1.9) | 3 (2.1) |  |
| Raised JVP or CXR signs (%) | 5 (2.3) | 2 (1.4) |  |
| Respiratory history |  |  | 0.9314 |
| No dyspnoea (%) | 173 (80.1) | 113 (80.7) |  |
| Dyspnoea on exertion (%) | 27 (12.5) | 19 (13.6) |  |
| Dyspnoea limiting exertion (%) | 11 (5.1) | 6 (4.3) |  |
| Dyspnoea at rest (%) | 5 (2.3) | 2 (1.4) |  |
| **Clinical values** |  |  |  |
| RDW (%) | 14 (13.2-15.2) | 13.8 (13.1-15) | 0.2957 |
| Haemoglobin (gl^-1^) | 131 (114-146) | 135 (120-146) | 0.3846 |
| Creatinine (μmol^l-1^) | 76 (66-100) | 75 (65-105) | 0.9248 |
| Urea (mmoll^-1^) | 5.8 (4.2-8.4) | 5.8 (4-9.6) | 0.6442 |
| Sodium (mmoll^-1^) | 138 (125-140) | 138 (135-140) | 0.9831 |
| WBC (x10^9^l^-1^) | 10.4 (7.5-15.4) | 10.3 (7.7-14.1) | 0.6922 |
| Systolic blood pressure  (mmHg) | 122 (110-138) | 126 (107-137) | 0.9633 |
| Pulse (beats min^-1^) | 87 (76-100) | 87 (74-102) | 0.8061 |
| **Perioperative** |  |  |  |
| Operative severity |  |  | 0.31 |
| Major (%) | 135 (62.5) | 95 (67.9) |  |
| Major+ (%) | 81 (37.5) | 45 (32.1) |  |
| Peritoneal soiling |  |  | 0.504 |
| None (%) | 98 (45.4) | 69 (49.3) |  |
| Serous fluid (%) | 55 (25.4) | 26 (18.6) |  |
| Localised pus (%) | 11 (5.1) | 8 (5.7) |  |
| Free bowel content, pus, or  blood (%) | 52 (24.1) | 37 (26.4) |  |
| Intraoperative blood loss |  |  | 0.03118 |
| <100ml (%) | 62 (28.7) | 60 (42.9) |  |
| 101-500ml (%) | 139 (64.4) | 72 (51.4) |  |
| 501-999ml (%) | 10 (4.6) | 7 (5) |  |
| >1000ml (%) | 5 (2.3) | 1 (0.7) |  |
| Severity of malignancy |  |  | 0.7418 |
| None (%) | 178 (82.4) | 122 (87.1) |  |
| Primary only (%) | 18 (8.3) | 9 (6.4) |  |
| Nodal metastasis (%) | 5 (2.3) | 2 (1.4) |  |
| Distant metastasis (%) | 15 (6.9) | 7 (5) |  |
| Observed 30-day mortality (%) | 16 (7.4) | 17 (12.1) | 0.139 |
| Observed overall mortality (%) | 44 (20.4) | 28 (20) | 1 |
| For 216 patient an assessment of frailty was either missing or not possible. Continuous variables are shown as median and interquartile ranges. Categorical variables are shown as a frequency (%). Non-winsorised values were used to draw up the table. P values were calculated using Wilcoxon-Mann-Whitney testing or the Chi square test with Yates continuity correction/Fisher’s exact test (testing for overall difference) as appropriate. AF: atrial fibrillation, ASA: American Society of Anaesthesiologist physical status classification system, CXR: chest radiograph, ECG: electrocardiogram, JVP: jugular venous pulse, Major+: all colonic resections, gastrectomy, laparostomy, intestinal bypass, reoperations for bleeding/sepsis, Major: all other including stoma formation, small bowel resection, adhesiolysis, repair of perforated/bleeding ulcer, NELA: National Emergency Laparotomy Audit, RDW: red cell distribution width, WBC: white blood cell count | | | |

Various approaches have been described to allow for missing data, depending on their missingness mechanism. Full information maximum likelihood estimation, multiple imputation and weighted adjustment methods are some of the techniques used [26]. However, when only the outcome variable has missing values, then complete case analysis is a valid choice [26].

Leaving out cases with missing frailty status, we fitted a logistic regression model for predicting frailty with RDW as a three-knot restricted cubic spline, logarithmic transformation of age, haemoglobin and sex. The model was oversimplified to reduce the effective degrees of freedom. In practice, it should contain in addition to the above predictors all other relevant variables such as comorbidities, functional ability and cognitive performance.

By prespecifying predictor complexity using measures of association to quantify the predictive potential for a variable (blinded to non-linearity) and shrinking model coefficients using penalised maximum likelihood estimation, we reduced model complexity further. The pentrace function in the rms package examines the Akaike Information Criterion (AIC) and the Schwarz Bayesian Information Criterion (BIC) for a variety of penalties so that the best cross-validating model can be chosen [9]. Shrinkage resulted in a reduction from 5 to 2.69 effective degrees freedom. Optimum penalty factors were 6.8 for simple terms and 125 for non-linear terms.

Similar to our survival analysis we checked the logistic regression model for multicollinearity, interactions and influential observations. Examining for collinearity, VIF values ranged between 1.03 and 22.65 (sex 1.03, age 1.06, haemoglobin 1.33, RDW spline 22.01, RDW 22.65). The global test of additivity (testing all interactions with RDW) had p= 0.4976, therefore we did not pursue interactions further. Screening for influential observations a total of 24 (17.1%) observations had a DFBETA≥$\pm$ 0.2. Of these, 7 (5%) cases were influential for RDW. Overall DFBETAS ranged from -0.69 to 0.55. We performed a sensitivity analysis removing the influential observations for RDW to determine their effect on the model. The overall χ^2^ increased from 15.11 to 17.08 and the RDW χ^2^ from 9.36 to 11.07, leading us to conclude that the effect of influential observations was minimal.

Our primary intention was to determine whether RDW is an independently associated with frailty. Although model validation is not strictly necessary for hypothesis testing, it quantifies the degree of overfitting and any other inaccuracies [19]. Our final model was validated using 400 bootstrap replications to estimate and then correct for optimism (Table 8). The fully penalised calibration curve is shown in Figure 8. A nonlinear calibration function estimate is particularly apparent at higher predicted risk.

| **Table 8**  Bootstrap (400 resamples) validation of fully penalised frailty model | | | | | | |
| --- | --- | --- | --- | --- | --- | --- |
| Index | Original sample | Training sample | Test  sample | Optimism | Corrected index | n |
| D_xy_ | 0.5316 | 0.5741 | 0.5049 | 0.0692 | 0.4624 | 400 |
| C-index | 0.7658 | 0.7871 | 0.7524 | 0.0347 | 0.7311 | 400 |
| R^2^ | 0.1673 | 0.1935 | 0.1800 | 0.0135 | 0.1538 | 400 |
| Intercept | 0.0000 | 0.0000 | 0.2254 | -0.2254 | 0.2254 | 400 |
| Slope | 1.0000 | 1.0000 | 1.1314 | -0.1314 | 1.1314 | 400 |
| Emax | 0.0000 | 0.0000 | 0.0663 | 0.0663 | 0.0663 | 400 |
| B | 0.1239 | 0.1176 | 0.1275 | -0.0100 | 0.1339 | 400 |
| D_xy_: Somers’ D_xy_ rank correlation, C-index: concordance probability (C=[D_xy_+1]/2), R^2^: Nagelkerke R^2^, slope & intercept needed to recalibrate the model equation to a 45° line, Emax: maximum absolute difference in predicted and calibrated probabilities, B: Brier quadratic probability score | | | | | | |


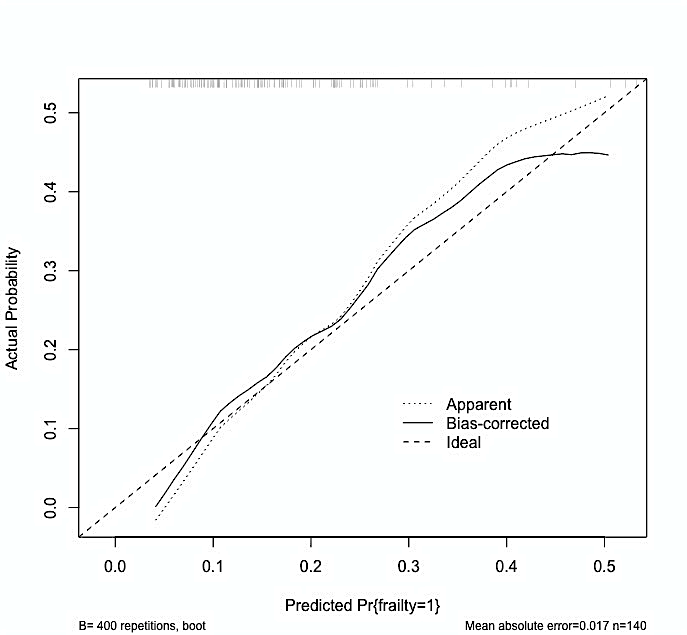


**Figure 8** Bootstrap calibration curve for the full penalised frailty model. 400 bootstrap repetitions were used in conjunction with the nonparametric *loess* smoother. A rug plot at the top of the graph illustrates the distribution of predicted risk. Mean absolute error= 0.017, mean squared error= 0.0005 and 0.9 Quantile of absolute error = 0.038 over the observed distribution of predicted values. Overfitting is apparent at higher predicted risks, where there is a scarcity of predicted values.

**References**:

1. R Core Team. R: A language and environment for statistical computing. R Foundation Statistical Computing, Vienna, Austria, 2018. <https://www.R-project.org/> (accessed 18/06/2018).

2. John Fox and Sanford Weisberg. An {R} Companion to Applied Regression, Second Edition. Thousand Oaks CA: Sage, 2011. <http://socserv.socsci.mcmaster.ca/jfox/Books/Companion> (accessed 18/06/2018).

3. Andri Signorell et al. DescTools: Tools for descriptive statistics. R package version 0.99.39, 2020. https://cran.r-project.org/web/packages/DescTools/DescTools.pdf (accessed 04/12/2020).

4. Hadley Wickham, Romain François, Lionel Henry and Kirill Müller. dplyr: A Grammar of Data Manipulation. R package version 0.8.0.1, 2019. <https://CRAN.R-project.org/package=dplyr> (accessed 27/05/2019).

5. H. Wickham. ggplot2: Elegant Graphics for Data Analysis. Springer-Verlag New York, 2016.

6. Mee Young Park and Trevor Hastie. glmpath: L1 Regularization Path for Generalized Linear Models and Cox Proportional Hazards Model. R package version 0.98, 2018. <https://CRAN.Rproject.org/package=glmpath> (accessed the 27/05/2019).

7. Baptiste Auguie. gridExtra: Miscellaneous Functions for "Grid"Graphics. R package version 2.3, 2017. <https://CRAN.R-project.org/package=gridExtra> (accessed 19/01/2019).

8. Frank E Harrell Jr, with contributions from Charles Dupont and many others. Hmisc: Harrell Miscellaneous. R package version 4.2-0, 2019. <https://CRAN.R-project.org/package=Hmisc> (accessed 03/02/2019).

9. Frank E Harrell Jr. rms: Regression Modelling Strategies. R package version 5.1-3, 2019.

<https://CRAN.R-project.org/package=rms> (accessed the 03/02/2019)

10. Therneau T. A Package for Survival Analysis in S. version 2.38, 2015.

<https://CRAN.R-project.org/package=survival> (accessed 28/01/2019)

11. Alboukadel Kassambara and Marcin Kosinski. survminer: Drawing Survival Curves using

'ggplot2'. R package version 0.4.3, 2018. <https://CRAN.R-project.org/package=survminer5> (accessed 28/01/2019).

12. National Emergency Laparotomy Audit. Audit inclusion and exclusion criteria, 2019. <https://www.nela.org.uk/Criteria> (accessed 20/03/2020).

13. National Emergency Laparotomy Audit. Patient information, 2014. <https://www.nela.org.uk/PatientInformation> (accessed 28 February 2019).

14. Eugene N, Oliver CM, Bassett MG, et al. Development and internal validation of a novel risk adjustment model for adult patients undergoing emergency laparotomy surgery: The National Emergency Laparotomy Audit risk model. *British Journal of Anaesthesia* 2018; **121**: 739-49.

15. Wood AM, White IR, Thompson SG. Are missing data adequately handled? A review of published randomised controlled trials in major medical journals. *Clinical Trials* 2004; **1**: 368-76.

16. Hewitt J, Carter B, McCarthy K, et al. Frailty predicts mortality in all emergency surgical admissions regardless of age. An observational study. *Age and Ageing* 2019; **48**:388-94.

17. Bland JM, Altman DG. The logrank test. *British Medical Journal* 2004; **328**: 1073.

18. Royston P, Choodari-Oskooei B, Parmar MKB, Rodgers JK. Combined test versus logrank/Cox test in 50 randomised trials. *Trials* 2019; **20**: 172.

19. Harrell FE. Regression modeling strategies, 2^nd^ edition. Switzerland: Springer International Publishing, 2015.

20. Croxford R. Restricted cubic spline regression: A brief introduction, 2016. <https://pdfs.semanticscholar.org/46d2/ffb174fe4b3e3091f3c24780f843877dfb7f.pdf> (accessed 10/02/2020).

21. Armitage P, Berry G, Matthews JNS. Statistical methods in medical research, 4^th^ edition. Oxford: Blackwell Science, 2002.

22. Kirkwood BR, Sterne JAC. Essential medical statistics, 2^nd^ edition. Oxford: Blackwell Science, 2003.

23. Harrell FE. Statistically efficient ways to quantify added predictive value of new measurements, 2019. <https://www.fharrell.com/post/addvalue/> (accessed 10/01/2020).

24. Pavlou M, Ambler G, Seaman SR, et al. How to develop a more accurate risk prediction model then there are few events. *British Medical Journal* 2015. Epub 11 August. doi.org/10.1136/bmj.h3868

25. James G, Witten D, Hastie T, Tibshirani R. An introduction to statistical learning with applications in R. New York: Springer, 2013.

26. Hughes RA, Heron J, Sterne JAC, Tilling K. Accounting for missing data in statistical analyses: multiple imputation is not always the answer. *International Journal of Epidemiology* 2019; **48**: 1294-304.
